# Perspectives from remote sensing to investigate the COVID-19 pandemic: A future-oriented approach

**DOI:** 10.3389/fpubh.2022.938811

**Published:** 2022-07-26

**Authors:** Khalid Mehmood, Yansong Bao, Sana Mushtaq, Saifullah, Muhammad Ajmal Khan, Nadeem Siddique, Muhammad Bilal, Zhang Heng, Li Huan, Muhammad Tariq, Sibtain Ahmad

**Affiliations:** ^1^Key Laboratory of Meteorological Disaster, Ministry of Education (KLME)/Joint International Research Laboratory of Climate and Environment Change (ILCEC)/Collaborative Innovation Center on Forecast and Evaluation of Meteorological Disasters (CIC-FEMD)/CMA Key Laboratory for Aerosol-Cloud-Precipitation Nanjing University of Information Science & Technology, Nanjing, China; ^2^School of Atmospheric Physics, Nanjing University of Information Science & Technology, Nanjing, China; ^3^School of Environmental Science and Engineering, Nanjing University of Information Science and Technology, Nanjing, China; ^4^Nishtar Medical University, Multan, Pakistan; ^5^Institute of Soil and Environmental Sciences, University of Agriculture, Faisalabad, Pakistan; ^6^Deanship of Library Affairs Imam Abdulrahman Bin Faisal University, Dammam, Saudi Arabia; ^7^Gad and Birgit Rausing Library, Lahore University of Management Sciences (LUMS), Lahore, Pakistan; ^8^School of Marine Sciences, Nanjing University of Information Science and Technology, Nanjing, China; ^9^Shanghai Satellite Engineering Institute, Shanghai, China; ^10^China Aerodynamics Research and Development Center, Mianyang, China; ^11^Department of Livestock Management, University of Agriculture, Sub-campus Toba Tek Singh, Faisalabad, Pakistan; ^12^Faculty of Animal Husbandry, Institute of Animal and Dairy Sciences, University of Agriculture, Faisalabad, Pakistan

**Keywords:** COVID-19, remote sensing, bibliometric analysis, visualization, network analysis

## Abstract

As scientific technology and space science progress, remote sensing has emerged as an innovative solution to ease the challenges of the COVID-19 pandemic. To examine the research characteristics and growth trends in using remote sensing for monitoring and managing the COVID-19 research, a bibliometric analysis was conducted on the scientific documents appearing in the Scopus database. A total of 1,509 documents on this study topic were indexed between 2020 and 2022, covering 165 countries, 577 journals, 5239 institutions, and 8,616 authors. The studies related to remote sensing and COVID-19 have a significant increase of 30% with 464 articles. The United States (429 articles, 28.42% of the global output), China (295 articles, 19.54% of the global output), and the United Kingdom (174 articles, 11.53%) appeared as the top three most contributions to the literature related to remote sensing and COVID-19 research. *Sustainability, Science of the Total Environment*, and *International Journal of Environmental Research and Public Health* were the three most productive journals in this research field. The utmost predominant themes were COVID-19, remote sensing, spatial analysis, coronavirus, lockdown, and air pollution. The expansion of these topics appears to be associated with cross-sectional research on remote sensing, evidence-based tools, satellite mapping, and geographic information systems (GIS). Global pandemic risks will be monitored and managed much more effectively in the coming years with the use of remote sensing technology.

## Introduction

Since the onset of severe acute respiratory syndrome coronavirus 2 (SARS-CoV-2) globally, the contributory factor of coronavirus disease 2019 (COVID-19), for more than 2 years, several combat strategies have been adopted over time. However, the infection level of the COVID-19 pandemic is alarming and thus the magnitude of the transmission has been enveloping the globe gradually (1, 2) and the World Health Organization (WHO) classifies the risk related to the new variant named “omicron” as very high (3, 4). COVID-19 has been associated with SARS-CoV-2 and was first reported from a seafood market in the city of Wuhan, China in December 2019, and later on, the world health organization (WHO), on March 11, 2020, declared COVID-19 as a global pandemic (5, 6). COVID-19 has infected nearly 523 million people with more than 6.2 million deaths globally (7). A large number of Asian countries, particularly those with large populations, have a high chance of being affected by this pandemic. This is due to the densities of the population, poverty, and a poor health care system in these countries (8, 9). Risk factors associated with COVID-19 lethality, pathogenicity, and rapid propagation of new emerging variants such as the delta variant are extremely intimidating and life-threatening for human health (10, 11). In many parts of the world, there is progressive ease in community interventions such as strict lockdowns and quarantines. Although worldwide vaccination has contributed significantly to decreasing COVID-19 cases, several disparities in health facilities and delayed access to vaccines could pose severe risks to successes achieved in controlling COVID-19 cases. Therefore, COVID-19 risk mitigation needs more coherent significant efforts to be eradicated from each territory irrespective of whether countries are developing or developed (12).

The contribution of remote sensing technology in tracing and fighting infectious diseases has been documented in the literature since 1694 (13). However, this was conducted by traditional mapping and this tool was used for plague control. Visualization and mapping analytics are continuously evolving in the health sector and disease control plan of every country that has resulted in the advancement of information technology and spatial science for infectious disease and epidemiology research (14, 15). In this scenario, space technologies are offering several optics of public and global health in terms of monitoring infectious disease, disasters, and climate change (14, 16). For example, space or satellite technologies offered satellite imagery to analyze the Ebola virus outbreak, disease spread, and emergency plans (14, 16). Therefore, the growing health burden with the improvements in different interfaces and tools offers visualizations of disease data in time and space (13, 17). In this context, satellite-based imageries offer an effective solution at both micro and macro scales to monitor the COVID-19 transmissions, community interventions, and other aspects related to the environment (1, 18). Several studies have been reported that are related to communities' exposure to different types of global and regional disasters (9, 19, 20). However, the case of the COVID-19 pandemic is different and complicated because it can easily transmit from one person to another person (21). This cross-sectional aspect of the COVID-19 pandemic warrants analysis which approves an interdisciplinary method. Earth Observation (EO) epidemiology is a pragmatic tool that is progressively being used by stakeholders and clinicians for zoonotic infections (14, 22, 23). EO epidemiology greatly helped in mapping out the transmission of the Ebola virus among animals and served as an innovative tool in risk communication, mapping, and categorizing vulnerable populations. For instance, Chen et al. (24) conducted a spatiotemporal analysis of traffic patterns through Planet Remote-Sensing Satellite Images during the period of COVID-19. Results suggested that high temporal planet data can provide enough traffic information for trend analysis that aids decision making during extreme phenomenon of COVID-19. Similarly, Minetto et al. (25) has observed changes in economic dynamics and population by applying satellite images before and during the COVID-19 pandemic over China, North Korea, the United States, Germany, and Russia. In addition, a growing study has employed remote sensing tools to estimate the air quality monitoring, and explored the impact of COVID-19 restrictions on air quality and it has grabbed the attention among scientific community as a breakthrough for air pollution mitigations efforts. For example, Ogen (26) examined the relationship between NO_2_ pollution and COVID-19 deaths through Sentinel-5P over Germany, Italy, France, and Spain. Results suggested that long-term exposure to NO_2_ pollution may increase the COVID-19 deaths in these regions.

Geography is one of the disciplines that provide an imitation approach to integrate both human and biophysical factors (27), by merging the environment from a holistic perception with an aim to forms and processes which harmonize in geographical space (28). Kost (29) reported the significance of point-of-care testing during COVID-19 pandemics using geospatial analysis for appropriate site selection globally. The authors concluded that the framework and monitoring of the COVID-19 pandemic are certainly required to adopt all available information and detect the areas or regions presently at high risk. Likewise, geographic information science (GIS) can increase the empathy and control of COVID-19 through observation, digital contact tracing, data sharing, and investigation of risk dynamics and infectious disease predictions (30, 31). Recently, China has developed different remote sensing and GIS applications to combat the COVID-19 pandemic especially the use of drones equipped with these techniques for perilous medical materials (32) and helped in identifying suitable sites for the construction of health amenities and the outbreak origin of COVID-19 (4, 33). From a health science point of view, the studies covering multiple variables of various types to understand the COVID-19 pandemic with spatiotemporal analysis and its geographical characteristics and disease evolution process are very important and a current necessity (9, 34). Recently, Zhou et al. (35) successfully employed GIS with a data mining approach by using spatiotemporal analysis and COVID-19 cases in China. Similarly, Rezaei et al. (36) also used spatiotemporal analysis with COVID-19 cases in Korea at provisional and national levels. Looking at the success and efficacy of these techniques to monitor and assess the COVID-19 pandemic, the utilization of statistical and geospatial technology is growing rapidly.

In this scenario, bibliometric methods and techniques offer an in-depth analysis of the tendencies and inspiration of its publications (37–40). It also depicts the development and emphases of different aspects in every field of merging science. So, in the scientific domain, bibliometric analysis has achieved substantial interest (41–43). This is the first study of its kind that improves our understanding of the contributions of remote sensing in the COVID-19 pandemic using comprehensive bibliometric analysis and explains the research prominence, elucidates ideas for scientists; and offers a guideline for research collaboration and implications of this work at a global scale. We covered the scientific production of articles that illustrated how remote sensing science was helpful during the COVID-19 pandemic by analyzing the productivity of the research in terms of different types of keyword analysis, statistical analysis, scientific journals metrics, and main authors publishing reports regarding the contribution of remote sensing tools in COVID-19. In addition, citations of works, impactful articles, countries, and institutions' research collaboration were analyzed to understand the role of remote sensing in the COVID-19 pandemic. Then, research articles were selected and categorized into different subject categories in terms of the most extensive studies on the subjects that developed substantial techniques and the most significant findings.

## Materials and methods

The current bibliometric study comprehensively analyzes scientific publications by constructing citation graphs and network analysis of different parameters such as author, countries, and research hotspots related to remote sensing and COVID-19. Scopus, one of the largest data sources (44, 45), was used to retrieve data. Scopus has been employed frequently for its quality standards, wide-ranging analysis in the assembly of data, and tremendous coverage of science journals (46). The current study carefully identified all possible keywords for maximum precision and recall. The keywords were connected with the use of Boolean Operators. The following search query was executed in the main search field of the data source Scopus on March 01, 2022.

TITLE-ABS-KEY (“remote sensing” OR “satellite imagery” OR “spatial analysis” OR “satellite data” OR “spatiotemporal analysis” OR “spatio-temporal analysis”) AND TITLE-ABS-KEY (coronavirus^*^ OR COVID-19 OR “COVID-19” OR COVID-19 OR ncov-^*^ OR hcov-^*^ OR sars-cov^*^ OR “ severe acute respiratory syndrome” OR mers-cov^*^ OR “Middle East Respiratory Syndrome” OR “corona virus”).

**Figure 1:**
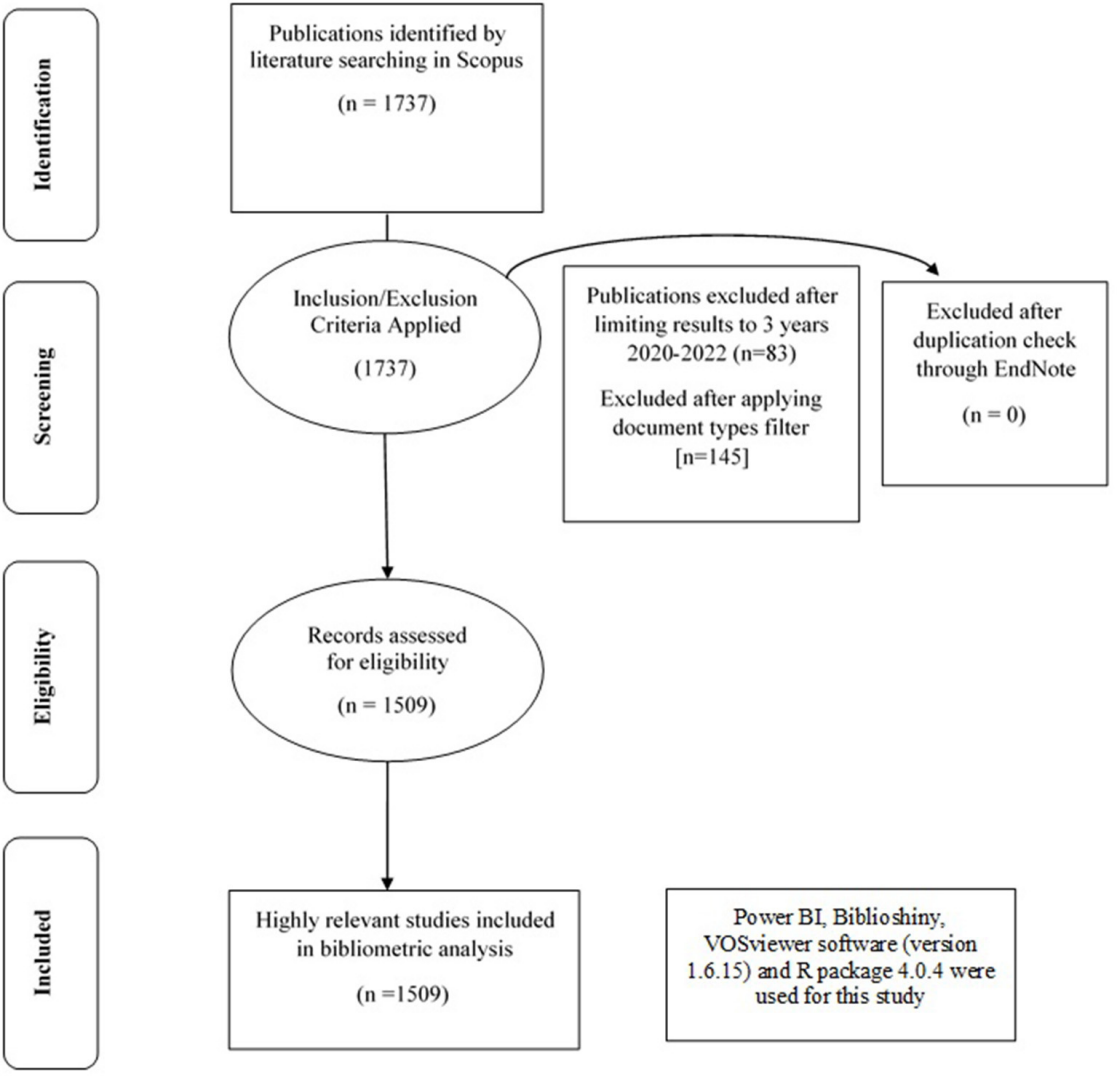
Four phase flow chart of data extraction and filtration process.

After applying search filters, the query finally resulted in 1,509 documents which were downloaded in RIS, CSV, and BIB formats. The data were imported into EndNote, a citation management software, to apply duplication check on the title, author, and year. No duplicate record was found. Then the final 1,509 documents, consisting of articles (1,327), book chapters (6), conference papers (67), and review articles (109) were used for the quantitative and qualitative analyses (Figure 1). This study employed exponential smoothing algorithms to predict total publications (TP) related to remote sensing and COVID-19. We also utilized correlation and regression analysis between TP and total citation (TC). *Analysis of Variance (ANOVA)* was performed for TP and within continents. *T-tests* were used to observe the difference between open and non-open access journals. The researchers used Power BI, Biblioshiny, MS Excel, MS Access, Biblioan Analytics, online visualization platform (https://flourish.studio/) for the data analysis. To prepare interactive plots, tabulations, and various kinds of visualization, we used VOSviewer software (version 1.6.15) and R package 4.0.4. Visualizations included country scientific collaboration, keyword co-occurrence, conceptual structure map, and three-field biplot. In order to display different types of items, including links, total link strength, and clusters, network, overlay, and density visualizations were applied. Cloud circles showed the magnitude of each variable (47). The *h-index* and *g-index* were used in this study. Researchers measure their productivity and citation impact using the h-index. Based on the number of citations an article received, the *g-index* is calculated for the set of articles. An additional variant of the *h-index* is the *m-index*, which indicates the published *h-index* per year since the document was first published.

## Results and discussions

### Publications growth and typology of documents

For the current study, we retrieved a total of 1,509 documents published in the English language, using the Scopus database covering the period from 2020 to 2022. As reported in other bibliometric studies (38, 48), the yielded documents were of 4 different types: the leading number of retrieved publications were research articles (1,327; 87.93%), followed by review papers (109; 7.22%), conference papers (67; 4.44%), and book chapters (6; 0.397%). The further analysis categorized the publications into access types, open access (OA) and not open access documents. Out of 1,509 publications, 1,280 publications were of OA type, and 229 were not openly accessible to the readers. In both categories, the document type “article” appeared as the most preferred document type adopted by the researchers for sharing their findings. Whereas, “conference paper” emerged as the second highly preferred document type in the not OA document category, “review articles” appeared as the second most preferred type in the OA category. Noteworthy, the document type “original article” secured the highest number of citations in both the categories, followed by “review.”

**Figure 2:**
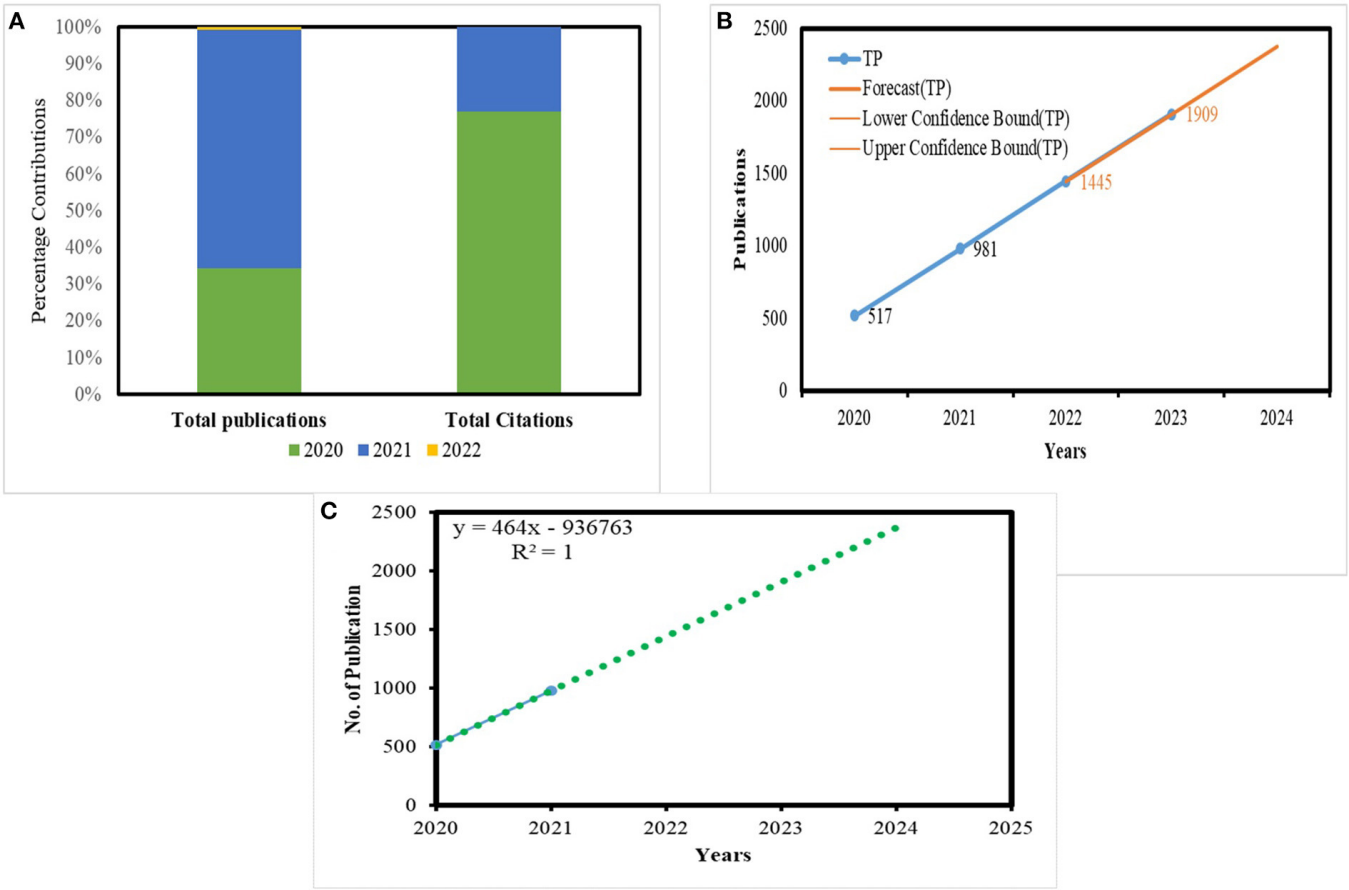
Stacked column analysis for remote sensing and COVID-19 research during 2020–2022 **(A)**. Total publication (TP) forecast with years **(B)**, and regression analysis of no. of publication **(C)**.

Also, we applied a 100% stacked column to compare the percentage contribution (%) of publications and citations between 2020 and 2022. A total of 517 documents with 34.26% contribution were published in 2020 and with a large number of 10,569 citations (Figure 2A). However, in the year 2021, the no. of documents increased to 981 documents with 65% contribution with only 3,141 citations. An extensive number of documents published in 2021 may be explained by the use of remote sensing techniques at a large scale during the COVID-19 pandemic. It also forecasts TP 1,445 and 1,909 for 2022 and 2023, respectively. Figures 2B,C are also provided for the same purpose. We also further analyzed the correlation between TP and total citations (TC) secured by the publications related to remote sensing. The analysis showed a significant correlation between TP and TC (Table 1). The value of pearson correlation shows a strong correlation between TP and TC. It can be inferred that the number of citations increases with the number of publications. A linear regression test was applied by taking TP as the independent variable and TC as the dependent variable to investigate the effect of the number of publications on the citations received.

**Table 1:** Statistical analysis (regression and correlation analysis, analysis of variance (ANOVA), and independent *t*-test) for different bibliometric variables used in this study.

	**Correlation analysis**				**Regression analysis**						
		**Total publication(TP)**	**Total citation 1**		**Variable**	**Unstandardized coefficients**	***p*-value**	**–**	**–**	**–**	
TP	Pearson correlation	1	0.980**		TP	11.413	0	–	–	–	
	Sig. (2-tailed)		0		R squared	0.96	–	–	–	–	
	*N*	120	120		a. Dependent variable: TC	–	–	–	–	–	
TC	Pearson correlation	0.980**	1		b. Predictors: (Constant), TP	–	–	–	–	–	
	Sig. (2-tailed)	0			–	–	–	–	–	–	
	*N*	120	120		–	–	–	–	–	–	
**Analysis of variance (ANOVA)**						**Analysis of variance (ANOVA)**					
**TP with continents**						**TC with continents**					
	**Sum of squares**	**Df**	**Mean square**	**F**	**Sig**.		**Sum of squares**	**df**	**Mean square**	**F**	**Sig**.
Between groups	108951.345	5	21790.3	11.483	0	Between groups	15,404,950	5	3,080,990	12.235	0
Within groups	216323.022	114	1897.57	–	–	Within groups	28,706,368	114	251810.2	–	–
Total	325274.367	119				Total	44,111,318	119	–	–	–
	***T*** **-test**					
	**Access type**	***N***	**Mean**	**Std. deviation**	**Std. error mean**						
TC	Non-open access	229	2.699	20.4665	1.3525	–	–	–	–	–	
	Open access	1,280	10.228	33.5103	0.9366	–	–	–	–	–	
		Levene's test for equality of variances		*t*-test for equality of means							
		***F***	**Sig**.	***T***	**Df**	**Sig. (2-tailed)**	**Mean difference**	**Std. error difference**	**95% confidence interval of the difference**		
									Lower	Upper	
TC	Equal variances assumed	17.898	0	−3.292	1,507	0.001	−7.5294	2.2875	−12.0164	−3.042	
	Equal variances not assumed	–		−4.577	479.49	0	−7.5294	1.6451	−10.762	−4.296	

The *p*-value of the linear regression shows a significant effect of TP on the TC. The positive value of unstandardized coefficients shows that the citations increase with an increase in publications (Table 1).

For example, El Mohadab et al. (49) analyzed the COVID-2019 pandemic by retrieving 10,228 documents from the Scopus database during May 2020, whereas the study of Zyoud and Al-Jabi (50), yielded 19,044 documents during June 2020 from the same database. This study implies that there is a sharp rise in publications related to COVID-19 aspects during 2020–2021 and that these studies contribute considerably to fostering the research and developments on COVID-19 that became a precedence agenda for scientists and decision-makers in various areas of science, especially spatial science. In addition, several other reasons associated with the sharp rise in the number of publications in a short window include a global pandemic which is impacting global health, researchers, and epidemiologist having enough time to document and distribute their studies due to lockdown in maximum countries, and the encouraging response from journals regarding COVID-19 work as an editorial choice (51). Another possible reason for a large number of publications with greater citations could be the invitations from journals and the publication of many special issues covering every aspect of the COVID-19 pandemic.

### Countries and institutions research related to COVID-19 and remote sensing

According to the first author affiliations of the work related to the contribution of remote sensing to the COVID-19 pandemic, a total of 165 countries with 5,239 institutions have participated in this subject area. The leading countries in the literature fetched from the Scopus database are the United States (429 articles, 28.42%), China (295 articles, 19.54%), the United Kingdom (174 articles, 11.53%), India (135 articles, 8.94%) and Spain (56, 3.71%). The *ANOVA* for the means of the TP within the continents. It was found that there is a significant difference in TP within the continents (*F* = 11.483, Sig. = 0.000).

**Figure 3:**
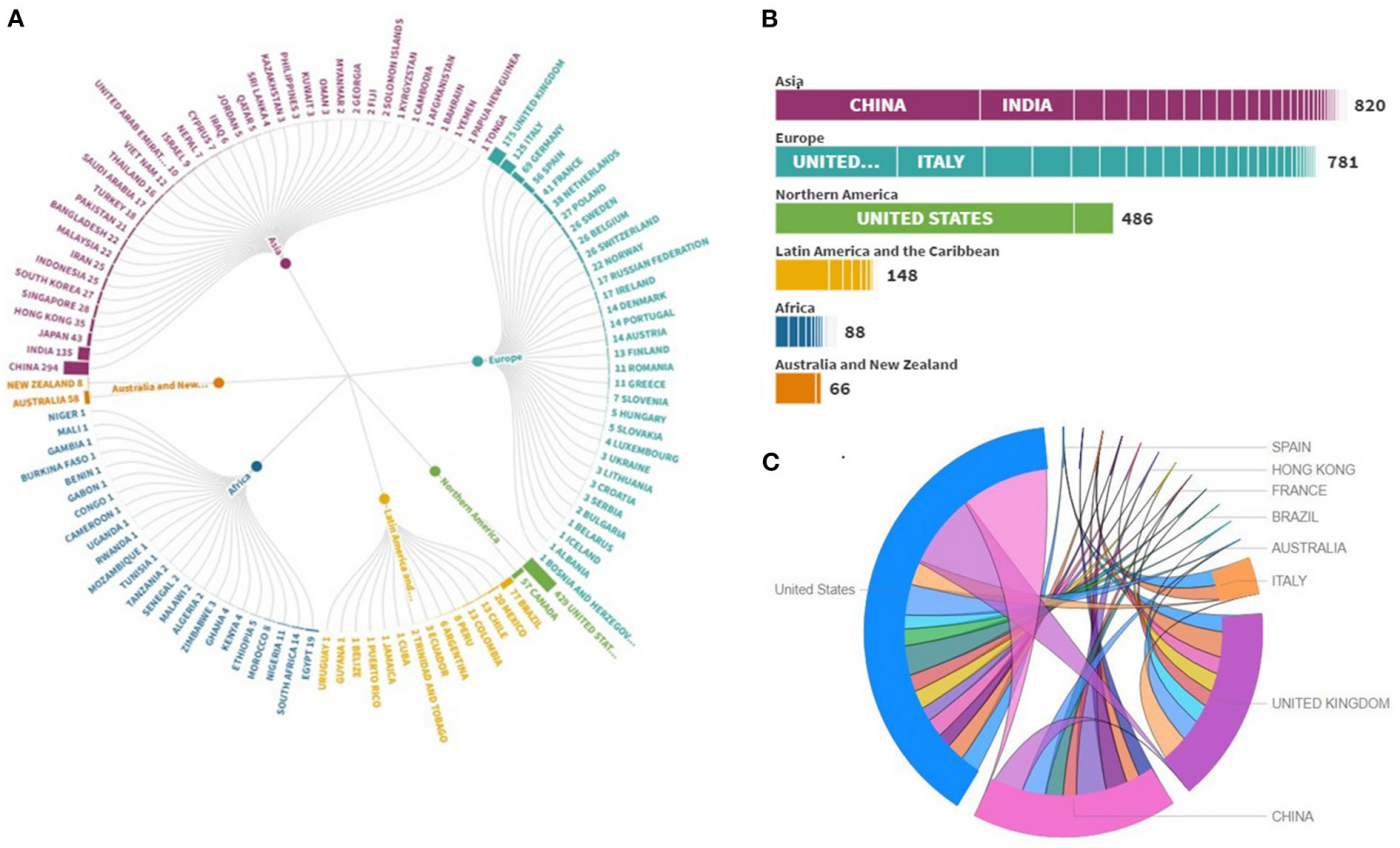
Research productivity of countries **(A)**, continent-wide breakdown of the publications emerging from the respective countries and continents **(B)**, key collaborations among other countries **(C)**.

In addition, the least significant difference (LSD) multiple comparisons as shown in Supplementary Table 1 indicates that only North America is significantly different from all other continents in terms of publications. Similarly, Table 1 shows the *ANOVA* for the means of the TC within the continents. The results show a significant difference in TC within the continents (*F* = 12.235, Sig. = 0.000), while the LSD multiple comparisons, as shown in Supplementary Table 2, indicate that only North America is significantly different from all other continents in terms of securing citation. The research productivity of countries on the topic has been illustrated in Figure 3A. The figure portrayed the publications emerging from relevant countries and continents as well. The USA emerged as the most productive country not only from North America but also globally. In comparison, China led the Asian countries and globally secured second position. In recent years, China's research productivity has focused on building world-class universities and first-class disciplines, aiming to build a number of elite universities and disciplines until 2050 and further enhance the quality of higher education. Compared to China, other Asian countries are far behind in terms of research productivity. This is maybe due to lack of research funding Similarly, the United Kingdom led the European countries and globally secured third position. Europe has developed over the past half-century a world-class modern higher education system with many elite universities, which has greatly facilitated world science and technology development. The sunburst chart also showed many countries all over the globe contributing research on the topic in a single-digit, including only one publication. Figure 3B further illustrates the continent-wide breakdown of the publications emerging from the respective countries and continents. Network analysis indicates that the United States has developed the strongest set-up having 5,853 citations and the total link strength is 961 followed by China (3,141 citations and 884 total link strength) and the United Kingdom (1,590 citations and 375 total link strength). The USA emerged on the chart as the most collaborative country in conducting joint research on the topic with the maximum number of countries in its network. Recently, COVID-19 EOdashboard (https://eodashboard.org/), has been developed by joint efforts between the US National Aeronautics and Space Administration (NASA), the European Space Agency (ESA), and the Japan Aerospace Exploration Agency, that monitor worldwide variations due to the COVID-19. The United Kingdom and China are the other two most prominent countries on the chart. It is apparent from the chart that the United States, the United Kingdom, and China have collaborated the maximum number of research studies with each other. Some other vital collaborations among other countries are also visible on the chart (Figure 3C).

Overall, the United States and China were the top two countries in which remote sensing techniques were used to fight the COVID-19 pandemic. For institutional participation in this subject area, we adopted the bibliographic coupling method to analyze their contribution in terms of total link strength and citations. Wuhan University has ranked first with total link strength of 6 and 26 citations followed by George Mason University with 5 total link strengths and 37 citations and California Institute of Technology with total link strength and 50 citations. It can be seen that the use of remote sensing for the COVID-19 pandemic is still limited in Asian countries except for China. The comprehensive overview of COVID-19 related to remote sensing demonstrates the excellent performance of developed countries.

### Author keywords and research hotspot analysis

**Figure 4:**
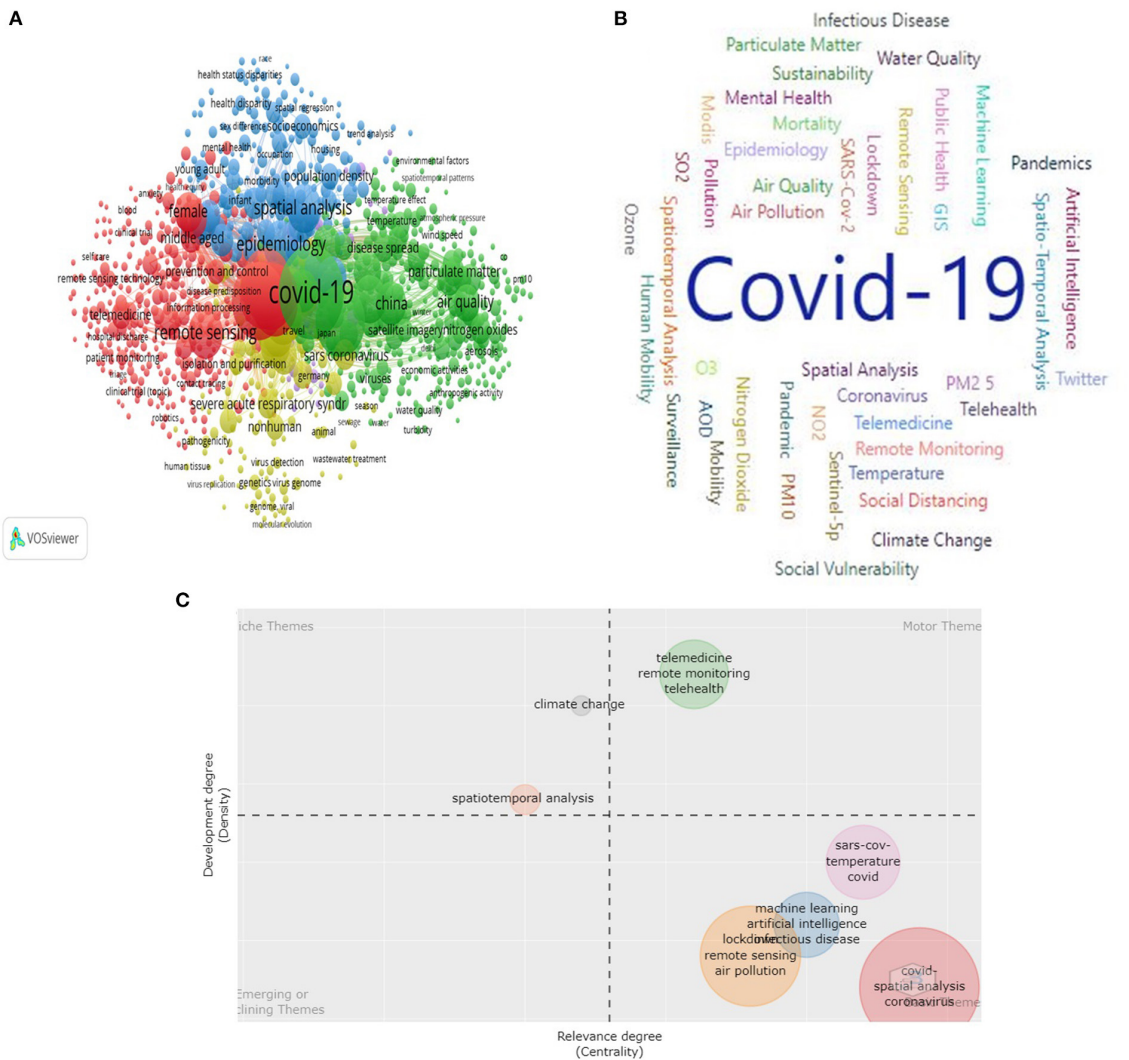
Author keywords network visualization **(A)**, word cloud map **(B)**, and thematic map **(C)**.

The author keywords illustrate a piece of information regarding keywords occurring in the article. This information is helpful, especially when exploring the research domain because they cover precise information that author wish to publish for their audience (52). This study showed the key milestones by analyzing the list of keywords that had strong links between 2020 and 2022, as seen in Figure 4A. The data revealed that the most recurrently occurred author keywords were “COVID-19” (11,147) with total link strength was 22,241, “remote sensing” (335) with total link strength of 4,451, “spatial analysis” (323) with total link strength of 4,585, and “spatiotemporal analysis” (132), with total link strength of 2050 entailing the significance of analyzing the contribution of remote sensing work during the COVID-19 pandemic (Figure 4A).

Figure 4B depicts the word cloud of author keywords. The thematic map of the author-supplied keywords (min. frequency = 5; the number of words = 500) helps to describe a map of the main themes of the topic under study (Figure 4C). Using the visualization software Biblioshiny, a map was developed by plotting the themes into four quadrants according to the centrality and density values along the axes. The clusters with the higher number of relevant documents have been discussed for the four quadrants. Motor themes (first quadrant): the themes that belong to the first quadrant (motor themes) are regarded as the themes with high centrality and density. These are well-developed and considered as the key themes to the research area. There are a few motor themes that consist of telemedicine, remote monitoring, and telehealth. Niche themes (second quadrant) are developed and specialized but not of central importance in the overall field. The theme of climate change appeared at the axes of the first quadrant, and spatiotemporal analysis appeared at the axes of the third quadrant. Peripheral themes (third quadrant) are regarded as underdeveloped and not centrally important, such as emerging and declining themes. No theme can be seen in this quadrant. Transversal and general, basic themes (fourth quadrant) are the themes that are still not well-developed, having high centrality and low density. The themes like COVID-19, spatial analysis, coronavirus, lockdown, remote sensing, and air pollution are the most recurring in this quadrant.

### Most productive sources

The analysis of journal productivity is useful to explore the information related to COVID-19 and remote sensing are published across journals and help the scholars to select the journal to disseminate their work. An independent *t*-test was applied to investigate the difference in securing citations between OA and non-open access journals. The results show a significant difference in securing citations between OA and non-open access journals (*F* = 17.898, Sig = 0.000). It is evident from the means that the OA journals have secured more citations than non-open access journals (Table 1). It was also noticed that most of the publications were documented in the environmental sciences domain (573 articles), followed by medicine (545 articles), and social sciences (332 articles). The journal “*Sustainability*” (IF_2020_;3.251) ranked at the top position with the contribution of 7.68% followed by “*Science of the Total Environment*” (IF_2020_;7.963) with a contribution of 4.17% and “*International Journal of Environmental Research and Public Health”* (IF_2020;_3.390) with the contribution of 3.57%. Table 2 highlights the most favorite outlets for publishing research related to remote sensing in terms of no. cited publication (NCP), cited publication (CP), TP, and TC. In contrast, the citation-wise analysis ranked “*Science of the Total Environment*” at the top position in securing the highest number of citations (2,492) keeping all sources far behind in securing citations. All other sources could get only <600 citations.

**Table 2:** Most productive journals/sources during 2020–2021.

**Sources**	**NCP**	**CP**	**TP**	**TC**
Sustainability	64	52	116	310
Science of the total environment	5	58	63	2492
International journal of environmental research and public health	13	41	54	366
Environmental research	10	30	40	591
PLoS one	15	20	35	136
Aerosol and air quality research	7	27	34	556
Remote sensing	11	17	28	153
Environmental science and pollution research	6	20	26	133
Nature communications	2	16	18	312
Air quality, atmosphere and health	3	14	17	251
Scientific reports	5	11	16	40
Environment, development and sustainability	2	12	14	95
Environmental research letters	5	8	13	139
Journal of medical internet research	3	9	12	80
Wuhan Daxue Xuebao (Xinxi Kexue Ban)/Geomatics and information science of Wuhan University	6	6	12	25
Health and place	1	11	12	87
Proceedings of the national academy of sciences of the United States of America	4	7	11	228
Geophysical research letters	2	9	11	152
Science	2	9	11	566
International journal of infectious diseases	3	8	11	174
Spatial and spatio-temporal epidemiology	5	6	11	99
Journal of environmental management	0	9	9	53

**Figure 5:**
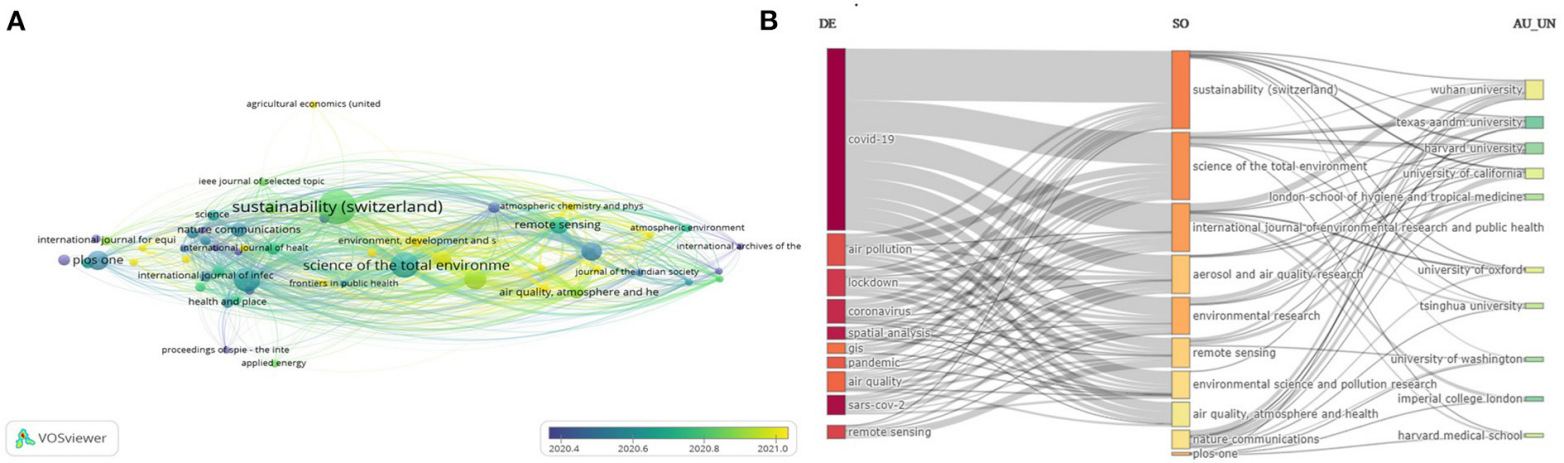
Most productive journal through overlay visualization (bibliographic coupling) with sources using full counting method **(A)**. Sankey three-field plot exploring relationship among keywords productive journals and institutions **(B)**.

The data also revealed cited and non-cited publications of the sources. The source “*Science of the Total Environment*” also leads all the sources in terms of the highest number of cited publications. Regarding the bibliographic coupling method, this study employed overlay visualization (full counting method) with sources and it is noticed that “*Science of the Total Environment”* has developed 6,525 total link strength followed by *Aerosol and Air Quality Research* with 4,928 total link strength and “*International Journal of Environmental Research and Public Health*” was found to have 4,928 total link strength (Figure 5A).

The highest contributor of papers related to COVID-19 and remote sensing was *Science of the Total Environment* because this journal has launched a special issue that was related to COVID-19 and grabbed the attention of the scientific community. A three field plot analysis, among author keywords (left), sources (middle), and organizations (right) has been shown in Figure 5B. The analysis highlights the author keyword COVID-19 as the most frequently used keyword that is published in almost all top sources, mostly in “*Sustainability*,” “*Science of the Total Environment*,” and “*International Journal of Environmental Research and Public Health*” and affiliated with organizations like “Wuhan University,” “Texas A&M University,” and “Harvard University.”

### Most productive authors with impact

**Figure 6:**
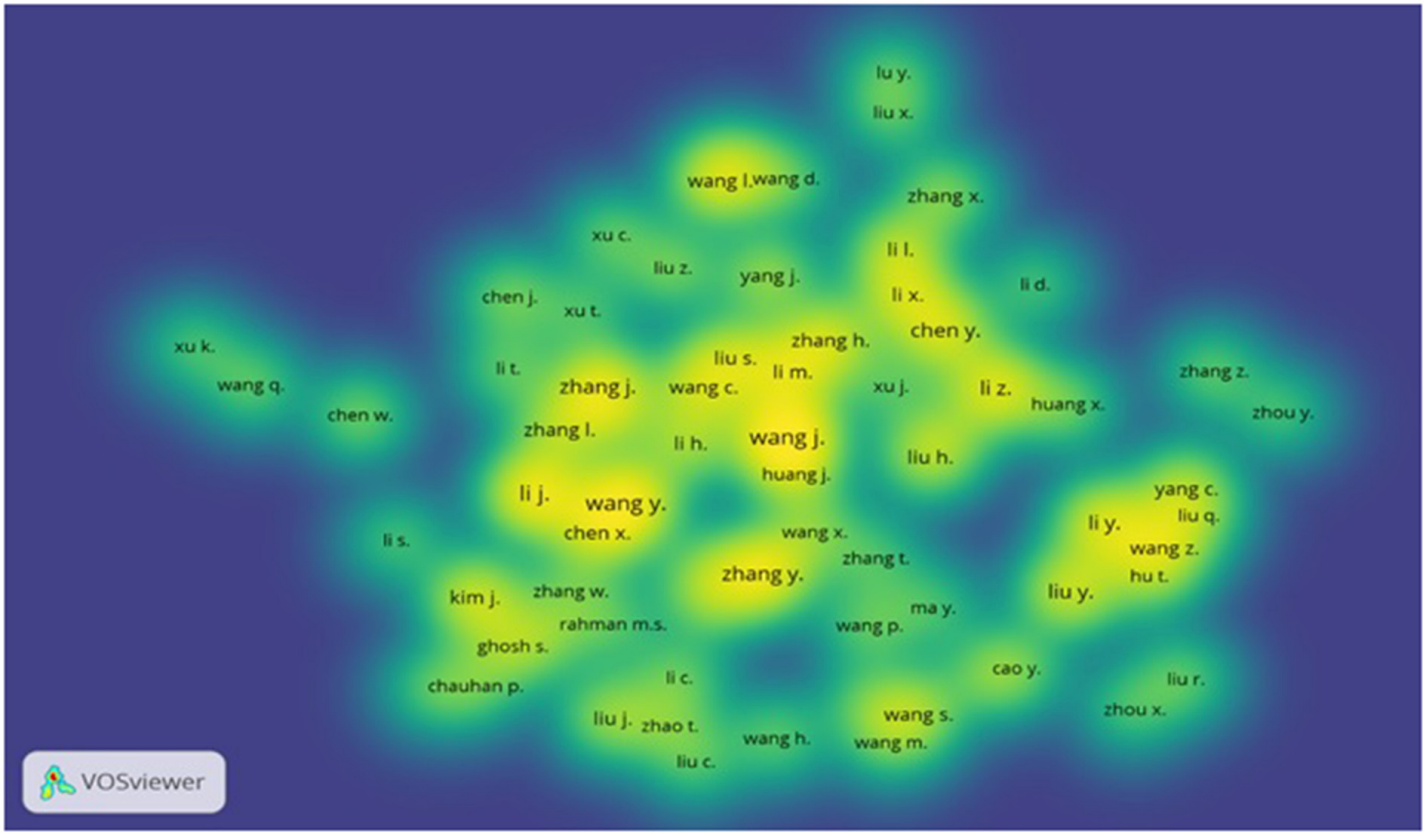
Density visualization of most productive authors.

Table 3 depicts the most productive authors with their impact. All authors in the table have been contributing research on the topic since 2020. The analysis ranked “Wang J” at the top position with 19 publications and 6 *h-index* and 17 *g-index*, followed by “Wang Y” with 14 publications and 8 *h-index* and 14 *g-index*. Two authors, “Zhang J” and “Zhang Y” remained at the third position due to contributing an equal number of publications. Likewise, the researchers “Li J” and “Li X” both maintained the fourth position due to publishing an equal number of documents. The second most prolific researcher, “Wang Y” secured the highest number of citations with a considerable margin from the topmost prolific author “Wang J.” The researcher “Wang H” obtained the second-highest number of citations although published only half of the documents as compared to “Wang Y.” Similarly, “Wang Y” achieved the highest *h-index* score among all authors. Whereas, “Wang J” emerged as the leading author in securing the highest *g-index* score. Similarly, the bibliographic coupling method suggested “Wang Y” has developed maximum total link strength (3,114) followed by “Wang J” (2,501) and “Zhang Y” (2,396; Figure 6).

**Table 3:** Most productive authors with metrics.

**Author**	**h_index**	**g_index**	**m_index**	**TC**	**NP**	**PY_start**
Wang J	6	17	3	305	19	2020
Wang Y	8	14	4	1,100	14	2020
Zhang J	5	13	2.5	236	13	2020
Zhang Y	4	9	2	88	13	2020
Li J	4	9	2	93	12	2020
Li X	3	4	1.5	34	12	2020
Kim J	6	8	3	75	11	2020
Li Z	3	10	1.5	115	11	2020
Li H	6	10	3	151	10	2020
Li Y	5	9	2.5	126	9	2020
Liu Y	3	6	1.5	42	9	2020
Chen Y	4	5	2	29	8	2020
Li M	5	8	2.5	94	8	2020
Wang L	4	7	2	62	8	2020
Chen J	5	7	2.5	218	7	2020
Liu J	4	7	2	75	7	2020
Liu S	4	6	2	45	7	2020
Wang C	3	5	1.5	33	7	2020
Wang H	5	7	2.5	816	7	2020
Zhang H	3	7	1.5	59	7	2020

We also applied co-citations analysis with cited authors using the full counting method. Results suggested “Wang Y” has gained 574 citations with 56,299 total link strength followed by “Zhang Y” with 317 citations and 31,923 total link strength and Liu Y with 348 citations and 27,334 total link strength.

### Top highly cited articles

The number of citation analysis designates its utmost key bibliometric landscapes since it displays the position and reputation of a study in the scientific community on a specific topic. The variable highly cited articles is a measure of high-quality publications in a particular field of research. The data pertaining to the most highly cited articles published on remote sensing and COVID-19 have been presented in Table 4. The article published by Ogen (26) secured the top position with a total of 349 citations during the current research period, distantly followed by Wang and Su (53) with 232 and Jia et al. (54) with 211 citations. The document Kanniah et al. (55), published in *Science of Total Environment* remained at the bottom of the list with a total of 113 citations. The top-ranked study conducted by Ogen Y has employed Sentinel-5P for mapping the tropospheric NO_2_ distribution and highlighted the role of NO_2_ in terms of deaths caused by the COVID-19 virus over Germany, Spain, France, and Italy. The second most impactful study conducted by Wang Q, also used Sentinel-5P data to analyze the positive impact of COVID-19 restrictions on the air quality. In the study of Jia S, they developed a spatio-temporal risk source model that influences population movement data not only to predict the distribution of COVID-19 positive cases but also to classify areas that have a high risk of spread at an emerging stage. Overall, these studies employed different satellite datasets and other spatiotemporal techniques for the purpose of studying the different aspects of COVID-19 that signify the use of spatiotemporal techniques.

**Table 4:** Highly cited articles during 2020–2022.

**References**	**Journal name**	**Doi**	**TC**	**TC per Year**	**Normalized TC**
Ogen (26)	Sci Total Environ	10.1016/j.scitotenv.2020.138605	349	174.5	17.0719
Wang and Su (53)	Sci Total Environ	10.1016/j.scitotenv.2020.138915	232	116	11.3487
Jia et al. (54)	Nature	10.1038/s41586-020-2284-y	211	105.5	10.3214
Mollalo et al. (58)	Sci Total Environ	10.1016/j.scitotenv.2020.138884	177	88.5	8.6582
Zhou et al. (35)	Geo Sustain	10.1016/j.geosus.2020.03.005	177	88.5	8.6582
Venter et al. (59)	Proc Natl Acad Sci USA	10.1073/pnas.2006853117	159	79.5	7.7777
Yunus et al. (60)	Sci Total Environ	10.1016/j.scitotenv.2020.139012	127	63.5	6.2124
Liu et al. (61)	Nat Commun	10.1038/s41467-020-18922-7	120	60	5.87
Kanga et al. (62)	Int J Infect Dis	10.1016/j.ijid.2020.03.076	119	59.5	5.8211
Kanniah et al. (55)	Sci Total Environ	10.1016/j.scitotenv.2020.139658	113	56.5	5.5276

## Contributions and implications of this work

With the beginning of the global COVID-19 pandemic, the rapid evolution of and alarming rise in infection of SARS-CoV-2 have enforced several countries to implement certain measures during 2020 and 2021 to minimize the further spread of the infection. These restrictions (complete lockdown, partial lockdown, and smart lockdown) have resulted in controlling the infection and have had diverse effects on the environment particularly in cluster cities and heavily populated regions due to a reduction in traffic mobility and industrial activities which ultimately reduced the pollution in the air, soil, and water. Infectious ailments usually acclimatize to anti-microbial and transmission characteristics and later turn into a global pandemic (56), so it is essential for public health authorities to recognize not only the disease prevalence but also its environmental variables and demographic patterns. In this context, disease incidence and environmental changes can be monitored by analyzing the spatio-temporal information and the vigorous spread of diseases simultaneously through remote sensing technology. In this study, we have selected some studies from our collected data based on their contributions and remote sensing techniques being employed in different aspects during pandemic during 2020–2021 (Table 5). It can be seen that a variety of different satellite products were used in the studies. However, the majority of the satellite sensors were used to monitor air quality especially during lockdown periods, for hotspot area identifications, and ecological studies (57). This study analysis revealed that this is also a helpful tool to monitor air pollution and can cover other environmental aspects in the future especially in the event of an unexpected pandemic outbreak. Results of the current study are promising, though there are still questions to be answered. A few limitations are present. In the first place, there is relatively little literature available in this area; therefore, bibliometric analyses may not report all aspects of this field's research. Additionally, the software design makes it more difficult to visualize the results of newly published high-level studies than older studies. Our goal is to improve upon these deficiencies and increase trend prediction accuracy in the future.

**Table 5:** Contributions of satellite techniques during COVID-19 pandemic 2020–2021.

**References**	**Study region/country**	**Remote sensing techniques/products**	**Contributions**	**Subject category**
Minetto et al. (25)	China, North Korea, USA, Germany, and Russia	The Intelligence Advanced Research Projects Activity (IARPA) Function Map of the World (FMoW) dataset.	This study analyzed the variations in economic variables and population dimensions.	Health and social geography
Kanga et al. (63)	India (Ramganj, Jaipur)	High-resolution satellite imagery from World View-1 (0.5 m) and GIS	This work designed a strategy to manage the spread of disease and highlighted risk zones.	Health and social geography
Kanga et al. (62)	India (Jaipur)	Worldview satellite imagery used for land-use/land cover (LULC) and GIS	This study designed a risk-based infrastructure to analyze the disease spread pattern and help the identification of hotspot areas.	Health and social geography
Chen et al. (24)	China (Wuhan), Japan (Tokyo), Rome, USA (New York), and India (New Delhi)	Vehicle Detection through Planet Remote-Sensing Satellite Images	Spatio-temporal analyses were conducted for the purpose to detect the traffic density and mobility during COVID-19 on a global scale.	Health and social geography
van Zyl and Celik (64)	Africa, Euro-Asia, and America	ESA Sentinel-1 constellation and Synthetic Aperture Radar (SAR)	This work monitored the reduction in human mobility enhances the human waste production with change in human activities	Environmental assessment
Elshorbany et al. (65)	United States (New York, California, Florida, Illinois, and Texas)	Ozone monitoring instrument (OMI) instrument aboard Aura Satellite, Measurement of Pollution in the Troposphere (MOPITT) instrument aboard Terra Satellite, Moderate Resolution Imaging Spectroradiometer (MODIS) Tera Collection, and Ozone Mapping Profiler Suite (OMPS) nadir-mapper (NM) v2.1	Examined the impacts of lockdown on air quality over different cities of the USA by using different satellite products.	Environmental assessment
Metya et al. (66)	China and India	OMI and Atmospheric Infrared Sounder (AIRS)	This study analyzes the air quality by using CO, NO2, and SO2 satellites during the outbreak	Environmental assessment
Ghasempour et al. (18)	Turkey	The TROPOspheric Monitoring Instrument (TROPOMI) and MODIS	This study examined the Spatiotemporal distributions density of SO2 and NO2 using satellite products and Google Earth Engine.	Environmental assessment
Ali et al. (67)	Multiple cities of Pakistan	TROPOMI, MODIS product (MCD19A2-V), Terra MODIS daily night-time LST composites (MOD11A2) at 1 km resolution.	Restrictions on transportation in multiple cities caused an obvious decrease in the surface urban heat island effect by using different satellite products, especially in megacities.	Environmental assessment
Sun et al. (68)	Wuhan (China)	Landsat-8/OLI, Sentinel-2/MSI, and HY-1C/CZI	This study conducted multi-sensor satellite images to estimate the turbidity of lakes)	Environmental assessment
**Geospatial dashboard application**	
Earth Observatory (EO)^a^	GEO Community Response to COVID-19	Multiple satellite products	This platform is offering research applications of Earth observations to advance understanding of COVID-19 transmission.	Web-based mapping
WHO Coronavirus Disease (COVID-19)^b^	Global	WHO's official data	This dashboard offers visualization and exploratory data analysis in terms of COVID-19 cases, and death counts by applying a 3D graph for each country.	Web-based mapping
Johns Hopkins University COVID-19^c^	Global	European Centre for Disease Prevention and Control (ECDC), US-CDC, and WorldoMeters	This is also used for visualizing COVID-19 daily cases data and COVID-19 waves and trend analysis.	Web-based mapping
Seismic Risk Map for COVID-19^b^	Global	COVID-19 data globally; Earthquake risk map; Global Earthquake Model (Source: GEM; JHU CSSE)	This database helps to Visualize the earthquake as a cause of an increase in COVID-19 cases which attributed to people's migration from damaged buildings	Web-based mapping

a *https://earthobservations.org/covid19.php (accessed March 15)*.

b *https://maps.openquake.org (accessed March 15)*.

c *https://coronavirus.jhu.edu/map.html (accessed March 15)*.

## Conclusions and discussion

The purpose of the present paper is to identify hidden knowledge underlying this significant body of research by retrieving relevant literature on remote sensing COVID-19. This study employed a comprehensive bibliometric analysis (based on 1,509 documents from the Scopus database) on the evolution and progress of remote sensing use in COVID-19 related studies. A part of this study involved selecting and categorizing research articles into different subjects based on the most extensive studies and important findings reported in those studies. Several bibliometric indices were utilized to uncover the research characteristics on the topic of remote sensing and COVID-19, concerning document topics, country productivity and institutional collaborations, active sources, keywords analysis, and most productive authors. Extensive documents (*n* = 981) were published in 2021, however, most citations (10,569) were grabbed in the year 2020. TP has a significant effect on TC, as indicated by the *p*-value of the linear regression. Unstandardized coefficients illustrate how publications increase the number of citations. The United States was found to be the most productive country in the world on this topic. In terms of citations secured, only North America stands out significantly from all other continents, according to the multiple comparisons of LSD. The most recurrently occurring author keywords were “COVID-19” (11,147), “remote sensing” (335), “spatial analysis” (323), and “spatiotemporal analysis” (132). According to author analysis, ranked “Wang J” at the top position with 19 publications and 6 *h-index* and 17 g-index, followed by “Wang Y” with 14 publications and 8 *h-index* and 14 g-index. The impactful article was published by Ogen (26) with a total of 349 citations. This compressive bibliometric work offers several important implications in terms of scientific productivity on the role of remote sensing and COVID-19. These analyses displayed several key information that can help researchers and decision-makers to acquire knowledge on the productivity of the countries, hotspots area of research, authors metric, and institutions on the theme of remote sensing and COVID-19. The cutting-edge research and research hotspot collected from literature offers detailed information on the current and future perspectives on this theme. The current study backed by an extensive analysis provides in-depth information for each parameter being used in this study. For instance, a country's scientific productivity implies the research status of a particular country in the future. Similarly, a highly cited article indicates the significance of the research for a specific topic of interest. These can be referred to other bibliometric parameters such as collaborations, no. of citation, etc. In addition, this study has selected and categorized research articles into different subjects based on the most extensive studies that developed substantial techniques and the most significant findings. So, the demarcation of tentative changes in the research domain and trend compared to the existing literature is an auxiliary key implication of this analysis. Further, COVID-19 research is at an emergent stage, the situation is changing rapidly over time due to new expected COVID-19 waves or new variants in the world in response to environmental changes and other unexplored factors. It can be deduced that remote sensing research on COVID-19 will certainly be helpful to introduce a method to advance access to space techniques by relevant stakeholders, in terms of satellite images to support numerous characteristics of the pandemic, such as environmental variables, and to the provision of medicine and vaccines and solving supply-chain problems. Development in remote sensing techniques could play a crucial role in the monitoring and prevention *via* the use of datasets for pandemic risk assessment modeling and several other factors of environment, ecology, and climate.

## Data availability statement

The original contributions presented in the study are included in the article/Supplementary material, further inquiries can be directed to the corresponding author/s.

## Author contributions

KM conducted the review and prepared the manuscript and wrote the initial draft of the manuscript and performed the literature research. YB and S are the guarantor of this study, designed the idea, and validated data. All authors read and approved the final manuscript. All authors agreed to be accountable for all aspects of the work in ensuring that questions related to the accuracy or integrity of any part of the work are appropriately investigated and resolved.

## Funding

This study was supported by Shanghai Aerospace Science and Technology Innovation Foundation under Grant No. SAST2020-032.

## Conflict of interest

The authors declare that the research was conducted in the absence of any commercial or financial relationships that could be construed as a potential conflict of interest.

## Publisher's note

All claims expressed in this article are solely those of the authors and do not necessarily represent those of their affiliated organizations, or those of the publisher, the editors and the reviewers. Any product that may be evaluated in this article, or claim that may be made by its manufacturer, is not guaranteed or endorsed by the publisher.

## Abbreviations

WHO: World Health Organization
NASA: National Aeronautics and Space Administration
EO: earth observatory
GIS: geographic information system
ANOVA: analysis of variance
TC: total citation
TP: total publication
OA: open access
NCP: no cited publication
CP: cited publication
IARPA: intelligence advanced research projects activity
FMoW: function map of the world
LULC: land-use/land cover
ESA: European Space Agency
OMI: ozone monitoring instrument
MOPITT: measurement of pollution in the troposphere
MODIS: moderate resolution imaging spectroradiometer
AIRS: atmospheric infrared sounder
TROPOMI: TROPOspheric monitoring instrument.
