# Supplementary material for: Perspectives from remote sensing to investigate the COVID-19 pandemic: A future-oriented approach
Source: Front Public Health. 2022 Jul 26;10:938811. doi: 10.3389/fpubh.2022.938811 (PMC9360797; doi:10.3389/fpubh.2022.938811)
Supplement: Supplementary file 1 [file Table_1.DOCX]

**Supplementary Information**

**Table S1.** Least significant difference (LSD) for total publications (TP) and continents ((Dependent variable: TC)

| **Multiple Comparisons** | | | | | | | | | |  |
| --- | --- | --- | --- | --- | --- | --- | --- | --- | --- | --- |
| (I) Continent_Code | (J) Continent_Code | Mean Difference (I-J) | Std. Error | Sig. | | 95% Confidence Interval | | | |  |
|  |  |  |  |  |  | Lower Bound | | Upper Bound | |  |
| Asia | Africa | 16.7051 | 10.8703 | .127 | | -4.829 | | 38.239 | |  |
|  | Australia | -12.8333 | 31.5272 | .685 | | -75.289 | | 49.622 | |  |
|  | Europe | -3.9242 | 10.1332 | .699 | | -23.998 | | 16.150 | |  |
|  | North America | -222.8333^*^ | 31.5272 | .000 | | -285.289 | | -160.378 | |  |
|  | South America | 10.1667 | 13.1029 | .439 | | -15.790 | | 36.123 | |  |
| Africa | Asia | -16.7051 | 10.8703 | .127 | | -38.239 | | 4.829 | |  |
|  | Australia | -29.5385 | 31.9651 | .357 | | -92.861 | | 33.784 | |  |
|  | Europe | -20.6294 | 11.4230 | .074 | | -43.258 | | 2.000 | |  |
|  | North America | -239.5385^*^ | 31.9651 | .000 | | -302.861 | | -176.216 | |  |
|  | South America | -6.5385 | 14.1240 | .644 | | -34.518 | | 21.441 | |  |
| Australia | Asia | 12.8333 | 31.5272 | .685 | | -49.622 | | 75.289 | |  |
|  | Africa | 29.5385 | 31.9651 | .357 | | -33.784 | | 92.861 | |  |
|  | Europe | 8.9091 | 31.7220 | .779 | | -53.932 | | 71.750 | |  |
|  | North America | -210.0000^*^ | 43.5611 | .000 | | -296.294 | | -123.706 | |  |
|  | South America | 23.0000 | 32.7916 | .484 | | -41.960 | | 87.960 | |  |
| Europe | Asia | 3.9242 | 10.1332 | .699 | | -16.150 | | 23.998 | |  |
|  | Africa | 20.6294 | 11.4230 | .074 | | -2.000 | | 43.258 | |  |
|  | Australia | -8.9091 | 31.7220 | .779 | | -71.750 | | 53.932 | |  |
|  | North America | -218.9091^*^ | 31.7220 | .000 | | -281.750 | | -156.068 | |  |
|  | South America | 14.0909 | 13.5649 | .301 | | -12.781 | | 40.963 | |  |
| North America | Asia | 222.8333^*^ | 31.5272 | .000 | | 160.378 | | 285.289 | |  |
|  | Africa | 239.5385^*^ | 31.9651 | .000 | | 176.216 | | 302.861 | |  |
|  | Australia | 210.0000^*^ | 43.5611 | .000 | | 123.706 | | 296.294 | |  |
|  | Europe | 218.9091^*^ | 31.7220 | .000 | | 156.068 | | 281.750 | |  |
|  | South America | 233.0000^*^ | 32.7916 | .000 | | 168.040 | | 297.960 | |  |
| South America | Asia | -10.1667 | 13.1029 | .439 | | -36.123 | | 15.790 | |  |
|  | Africa | 6.5385 | 14.1240 | .644 | | -21.441 | | 34.518 | |  |
|  | Australia | -23.0000 | 32.7916 | .484 | | -87.960 | | 41.960 | |  |
|  | Europe | -14.0909 | 13.5649 | .301 | | -40.963 | | 12.781 | |  |
|  | North America | -233.0000^*^ | 32.7916 | .000 | | -297.960 | | -168.040 | |  |
| *. The mean difference is significant at the 0.05 level. | | | | | | | | | |  |
| **Table S2.** Least significant difference (LSD) for total citation (TC) and continents (Dependent variable: TC) | | | | | | | | | | |
| **Multiple Comparisons** | | | | | | | | | | |
| (I) Continent_Code | (J) Continent_Code | Mean Difference (I-J) | Std. Error | | Sig. | | 95% Confidence Interval | | | |
|  |  |  |  |  |  |  | Lower Bound | | Upper Bound | |
| Asia | Africa | 177.4212 | 125.2218 | | .159 | | -70.642 | | 425.485 | |
|  | Australia | -109.3095 | 363.1812 | | .764 | | -828.769 | | 610.150 | |
|  | Europe | -73.6580 | 116.7309 | | .529 | | -304.901 | | 157.585 | |
|  | North America | -2654.3095^*^ | 363.1812 | | .000 | | -3373.769 | | -1934.850 | |
|  | South America | 142.5905 | 150.9398 | | .347 | | -156.420 | | 441.601 | |
| Africa | Asia | -177.4212 | 125.2218 | | .159 | | -425.485 | | 70.642 | |
|  | Australia | -286.7308 | 368.2257 | | .438 | | -1016.183 | | 442.721 | |
|  | Europe | -251.0793 | 131.5888 | | .059 | | -511.756 | | 9.597 | |
|  | North America | -2831.7308^*^ | 368.2257 | | .000 | | -3561.183 | | -2102.279 | |
|  | South America | -34.8308 | 162.7033 | | .831 | | -357.145 | | 287.483 | |
| Australia | Asia | 109.3095 | 363.1812 | | .764 | | -610.150 | | 828.769 | |
|  | Africa | 286.7308 | 368.2257 | | .438 | | -442.721 | | 1016.183 | |
|  | Europe | 35.6515 | 365.4254 | | .922 | | -688.253 | | 759.556 | |
|  | North America | -2545.0000^*^ | 501.8070 | | .000 | | -3539.076 | | -1550.924 | |
|  | South America | 251.9000 | 377.7466 | | .506 | | -496.413 | | 1000.213 | |
| Europe | Asia | 73.6580 | 116.7309 | | .529 | | -157.585 | | 304.901 | |
|  | Africa | 251.0793 | 131.5888 | | .059 | | -9.597 | | 511.756 | |
|  | Australia | -35.6515 | 365.4254 | | .922 | | -759.556 | | 688.253 | |
|  | North America | -2580.6515^*^ | 365.4254 | | .000 | | -3304.556 | | -1856.747 | |
|  | South America | 216.2485 | 156.2625 | | .169 | | -93.306 | | 525.803 | |
| North America | Asia | 2654.3095^*^ | 363.1812 | | .000 | | 1934.850 | | 3373.769 | |
|  | Africa | 2831.7308^*^ | 368.2257 | | .000 | | 2102.279 | | 3561.183 | |
|  | Australia | 2545.0000^*^ | 501.8070 | | .000 | | 1550.924 | | 3539.076 | |
|  | Europe | 2580.6515^*^ | 365.4254 | | .000 | | 1856.747 | | 3304.556 | |
|  | South America | 2796.9000^*^ | 377.7466 | | .000 | | 2048.587 | | 3545.213 | |
| South America | Asia | -142.5905 | 150.9398 | | .347 | | -441.601 | | 156.420 | |
|  | Africa | 34.8308 | 162.7033 | | .831 | | -287.483 | | 357.145 | |
|  | Australia | -251.9000 | 377.7466 | | .506 | | -1000.213 | | 496.413 | |
|  | Europe | -216.2485 | 156.2625 | | .169 | | -525.803 | | 93.306 | |
|  | North America | -2796.9000^*^ | 377.7466 | | .000 | | -3545.213 | | -2048.587 | |
| *. The mean difference is significant at the 0.05 level. | | | | | | | | | | |
